# H5N1 Influenza A Virus and Infected Human Plasma

**DOI:** 10.3201/eid1206.060227

**Published:** 2006-06

**Authors:** Salin Chutinimitkul, Parvapan Bhattarakosol, Surangrat Srisuratanon, Atthapon Eiamudomkan, Kittipong Kongsomboon, Sudarat Damrongwatanapokin, Arunee Chaisingh, Kamol Suwannakarn, Thaweesak Chieochansin, Apiradee Theamboonlers, Yong Poovorawan

**Affiliations:** * Chulalongkorn University Bangkok, Bangkok, Thailand;; † Srinakharinwirot University, Nakhon Nayok, Thailand;; ‡ National Institute of Animal Health, Bangkok, Thailand

**Keywords:** H5N1, influenza A virus, human, plasma, letter

**To the Editor:** Since January 2004, a total of 22 persons have been confirmed infected with avian influenza A virus (H5N1) in Thailand; 14 of these patients died. Three waves of outbreaks occurred during the past 2 years. The last patient of the third wave was a 5-year-old boy whose symptoms developed on November 28, 2005; he was hospitalized on December 5 and died 2 days later. The child resided in the Ongkharak District, Nakhon Nayok Province, ≈70 km northeast of Bangkok. Villagers informed the Department of Livestock after the patient's illness was diagnosed. Five dead chickens had been reported in this area from November 28 to December 1, 2005. Samples from these chickens could not be obtained, thus, no H5N1 testing was performed. The boy had fever, headache, and productive cough for 7 days before he was admitted to the Her Royal Highness Princess Maha Chakri Sirindhorn Medical Center. Clinical examination and chest radiograph showed evidence of lobar pneumonia. He was treated with antimicrobial drugs (midecamycin and penicillin G) and supportive care, including oxygen therapy. On December 7, the patient's condition worsened, and severe pneumonia with adult respiratory distress syndrome developed. Laboratory tests showed leukopenia (2,300 cells/mm^3^), acidosis, and low blood oxygen saturation by cutaneous pulse oximetry (81.6%). Oseltamivir was administered after his parents informed hospital staff about the boy's contact with the dead chicken. However, the boy died the same day; no autopsy was performed. On December 9, the cause of death was declared by the Ministry of Public Health to be H5N1 influenza virus.

A blood sample was collected from the patient on December 7; anticoagulation was accomplished with ethylenediaminetetraacetic acid (EDTA) for repeated biochemistry analysis and complete blood count. The plasma from the EDTA blood sample was separated 2 days later and stored at –20°C for 12 days. The sample was subsequently given to the Center of Excellence in Viral Hepatitis, Faculty of Medicine, Chulalongkorn University, for molecular diagnosis and then stored at –70°C, where specific precautions implemented for handling highly infectious disease specimens such as H5N1 influenza virus were observed. Plasma was examined by multiplex reverse transcription–polymerase chain reaction (RT-PCR) (*1*) and multiplex real-time RT-PCR (*2*), both of which showed positive results for H5N1 virus. The virus titer obtained from the plasma was 3.08 × 10^3^ copies/mL. The plasma specimen was processed for virus isolation by embryonated egg injection, according to the standard protocol described by Harmon (*3*). Briefly, 100 μL 1:2 diluted plasma was injected into the allantoic cavity of a 9-day-old embryonated egg and incubated at 37°C. The infected embryo died within 48 hours, and the allantoic fluid was shown to contain 2,048 hemagglutinin (HA) units; also, subtype H5N1 was confirmed (*1**,**2*). Whole genome sequencing was performed and submitted to the GenBank database under the strain A/Thailand/NK165/05 accession no. DQ 372591-8. The phylogenetic trees of the HA and neuraminidase (NA) genes were constructed by using MEGA 3 (*4*) for comparison with H5N1 viruses isolated from humans, tigers, and chickens from previous outbreaks in 2004 and 2005 (Figure). The sequence analyses of the viruses showed that the HA cleavage site contained SPQRE**K**RRKKR, which differed from the 2004 H5N1 virus by an arginine-to-lysine substitution at position 341. That finding had also been observed in wild bird species during earlier outbreaks in Thailand in 2004 (*5*). Similar to the 2004–2005 H5N1 isolates from Thailand, a 20–amino acid deletion at the NA stalk region was observed. Moreover, the amino acid residues (E119, H274, R292, and N294) of the NA active site were conserved, which suggests that the virus was sensitive to oseltamivir. In addition, a single amino acid substitution from glutamic acid to lysine at position 627 of PB2 showed increased virus replication efficiency in mammals (*6*).

**Figure:**
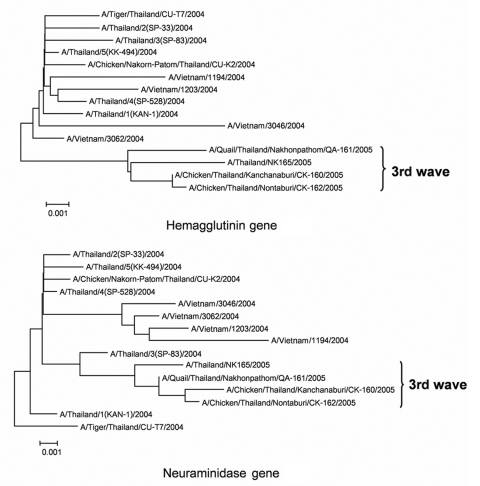
Phylogenetic analysis of the hemagglutinin and neuraminidase genes of H5N1 from study patient compared with sequences from previous outbreaks (2004–2005).

Observing live influenza virus in human serum or plasma is unusual. However, in 1963, low quantities of virus were isolated from blood of a patient on day 4 of illness (*7*), and in 1970, the virus was cultivated from blood specimens from 2 patients (*8*). Recently, a fatal case of avian influenza A (H5N1) in a Vietnamese child was reported. The diagnosis was determined by isolating the virus from cerebrospinal fluid, fecal, throat, and serum specimens (*9*); viral RNA was found in 6 of 7 serum specimens 4–9 days after the onset of illness (*10*). In this case, the H5N1 virus could be isolated from plasma on day 10 after symptoms developed. This case showed the virus in the patient's blood, which raises concern about transmission among humans. Because probable H5N1 avian influenza transmission among humans has been reported (*11*), this case should be a reminder of the necessity to carefully handle and transport serum or plasma samples suspected to be infected with H5N1 avian influenza. Because viable virus has been detected in blood samples, handling, transportation, and testing of blood samples should be performed in a biosafety (category III) containment laboratory to prevent the spread of the virus to healthcare and laboratory workers.

We express our thanks to the Thailand Research Fund (Senior Research Scholar), Royal Golden Jubilee PhD Program and Center of Excellence in Viral Hepatitis Research, and Prasert Auewarakul for their generous support of our study.
